# Cognitive behavioural therapy in virtual reality treatments across mental health conditions: a systematic review

**DOI:** 10.17650/2712-7672-2020-1-1-30-46

**Published:** 2020-09-02

**Authors:** Merve Dilgul, Jasmine Martinez, Neelam Laxhman, Stefan Priebe, Victoria Bird

**Affiliations:** Unit for Social and Community Psychiatry, Queen Mary University of London; East London NHS Foundation Trust

**Keywords:** Virtual Reality, Digital Interventions, Narrative Analysis, Mental Health Treatment, Cognitive Behavioural Therapy, виртуальная реальность, цифровые вмешательства, описательный анализ, терапия психических расстройств, когнитивно-поведенческая терапия

## Abstract

**Background.:**

Virtual reality (VR) has been effectively used in the treatment of many mental health disorders.However, significant gaps exist in the literature. There is no treatment framework for researchers to use when developing new VR treatments. One recommended treatment across a range of diagnoses, which may be suitable for use in VR treatments, is Cognitive Behavioural Therapy (CBT). The aim of this systematic review is to investigateCBT treatment methods that utilize VR to treat mental health disorders.

**Objectives.:**

To investigate how CBT has been used in VR to treat mental health disorders and to report onthe treatment characteristics (number of sessions, duration, and frequency) that are linked to effective and ineffective trials.

**Methods.:**

Studies were included if patients had a mental health diagnosis and their treatment included immersiveVR technology and CBT principles. Data were extracted in relation to treatment characteristics and outcomes,and analysed using narrative synthesis.

**Results.:**

Ninety-three studies were analysed. Exposure-based VR treatments were mainly used to treat anxiety related disorders. Treatments generally consisted of eight sessions, once a week for approximately one hour. VR treatments were commonly equal to or more effective than traditional face-to-face methods. No specific treatment characteristics were linked to this effectiveness.

**Conclusion.:**

The number, frequency and duration of the VR treatment sessions identified in this review, could be used as a treatment framework by researchers and clinicians. This could potentially save researchers time and money when developing new interventions.

## INTRODUCTION

Virtual reality (VR) is a technological interface that allows users to experience computer-generated environments within a controlled setting [1]. Recent meta-analyses and systematic reviews have found this technology to be an effective tool in the treatment of a range of mental health conditions [2], with most evidence derived from anxiety-related disorders [3], eating disorders [4] and psychosis [5].

In addition to its treatment effectiveness, VR exposure therapy has been found to be more cost-effective than face-to-face treatment for post-traumatic stress disorder [6]. Furthermore, VR treatments are well accepted by patients, who have expressed high levels of support and interest in its use for their mental health treatment [7]. There is also evidence that drop-out rates may be lower with VR treatments than with traditional face-to-face treatments [8]. This technology may, therefore, potentially improve access and adherence to psychological treatments [7,8].

Despite the potential of VR in mental health treatment, significant gaps exist in the literature relating to VR treatment. Studies in the literature have mainly focused on treating anxiety disorders with exposure-based therapies and have overlooked other diagnoses (e.g., depression, bipolar and personality disorder) and other treatment possibilities (e.g., guided self-help) [8].

A framework is a basic structure that underlies a system or concept, and may be built on or used as a point of reference to decide upon a particular course of action [9]. To our knowledge, there are no shared VR treatment frameworks currently available for researchers to follow. Without a treatment framework on which to build, researchers who want to explore new VR treatment methods for overlooked diagnoses, are forced to spend a great deal of time and money to develop their own treatments, which may or may not be successful [10]. The potential risks associated with not having a treatment framework, may constitute a barrier to new VR treatment methods.

One recommended treatment across a range of diagnoses [11], which may be suitable for use in VR treatments, is cognitive behavioural therapy (CBT). CBT is based on the cognitive model of mental illness and this model hypothesizes that the way in which patients feel and behave, is determined by their perception of situations, rather than the actual situations [12]. CBT aims to relieve distress by helping patients develop more adaptive cognitions and behaviours [13]. Developing a treatment framework that summarizes effective VR CBT treatment characteristics (e.g., the number of sessions, duration and frequency) could provide a possible foundation upon which researchers can build. This could potentially reduce the time and money spent on the development of interventions.

At present, no research has synthesized VR treatment characteristics across diagnoses. The aim of this systematic review is to explore CBT treatment methods that utilize VR to treat mental health disorders. A treatment framework will be developed from the identified shared treatment characteristics (e.g., the number of sessions, duration and frequency).

### Objectives

The objectives of this systematic review are to:

1. investigate how CBT has been used in VR to treat mental health disorders.
2. report on the treatment characteristics (number of sessions, duration, and frequency) that are linked to effective and ineffective trials.

## METHODS

The study protocol for this systematic review and narrative synthesis was registered on PROSPERO [CRD42018106757].

### Identification of studies

The eligibility criteria were developed using the PICO framework [14]. Papers were eligible if they were written in English, the study participants had to be over the age of 18 with any mental health diagnosis, using recognized diagnostic criteria (ICD-10 or DSM-V) or a validated scale with a pre-defined cut off point. To be included in the review, the interventions in the studies had to use principles of CBT, as defined by the NHS [15]. Furthermore, the VR technology used, had to be immersive. Immersive VR is defined as a computer-synthesized virtual environment surrounding the user. This can include (but is not restricted to) a head-mounted display (HMD) and a Cave automatic virtual environment (CAVE). An HMD consists of a computer generated video display attached to the user's head, with retina or head trackers that measure the changing position, which is fed back to the rendering computer. A CAVE is essentially a room in which computer generated visual imagery is projected onto the walls, floor and ceiling, and the user is free to move around. Papers were excluded if they did not have an experimental design (e.g., case series and reviews) and if the treatment procedures were not reported. All comparators and mental health-related outcomes were taken into consideration, including treatment effectiveness, feasibility, adherence and attrition.

A literature search of PubMed, CINAHL, EMBASE, PsycINFO, the Cochrane Library and NICE Healthcare Databases Advanced Search was conducted in August 2018. Grey literature was also searched using OpenGrey and Google Scholar. The search strategy was developed by identifying relevant key terms, used in a previous VR review [8] and was further developed in conjunction with an information scientist. The general search terms were: 'virtual reality' AND 'cognitive behavioural therapy' AND disorder-specific terms (see Appendix A for full search terms). Databases were searched from inception for titles, abstracts and keywords. Four key papers were identified and used to assess the reliability of the search results [1,8,16,17]. The authors also conducted hand searches of the Annual Review of CyberTherapy and Telemedicine and the reference list of relevant papers. Study authors were contacted when access issues occurred.

### Study selection

Identified references were transferred into Endnote and duplicates removed. The references were then transferred into an Excel spreadsheet. The first reviewer (MD) screened all the titles and abstracts, whereas the second reviewer (NL) independently screened 25%. Subsequently, the full text of the potentially relevant papers was retrieved and was once again independently assessed for eligibility by both reviewers. Hereafter, the reasons for exclusion were noted in the database. The inter-rater reliability for screening between the authors (MD and NL) using Cohen's Kappa was moderate (60% agreement, p<.0001). Any disagreements throughout the screening process were resolved through discussion and, if necessary, by involving a third reviewer (VB).

A data extraction framework was created using Excel and piloted with five studies. The data extracted included general information relating to the study eligibility, methods, VR treatment descriptions and a summary of the results, outcomes and conclusions.

Data were analysed using narrative synthesis [18]. Some treatment characteristics such as number and duration of sessions, were reported numerically, other treatment characteristics such as type of VR technology used and treatment location, were simplified into categorical variables for quantitative synthesis. This was to allow synthesis and integration of a large amount of data across the dataset. The quantitative data were imported into SPSS to allow for vote counting and for the statistical testing of differences. Vote counting and quantitative synthesis (e.g., t-tests and Chi-squared) were used to develop a preliminary synthesis, as they allowed the researchers to identify patterns across the included studies [18].

The first objective of this review was to investigate how CBT has been used in VR to treat mental health disorders. Once the treatment characteristics of all of the 93 studies were synthesized, the first objective of this review had been achieved.

The second objective was to report on the treatment characteristics that are linked to effective and ineffective trials. Studies which aimed to explore VR treatment effectiveness (62 out of the 93 studies) were selected for the second analysis. These studies were categorized according to their aims and were analysed separately.

Finally, treatment characteristics of studies which found VR to be more effective by comparison with 'traditional' treatment methods (e.g., in-vivo exposure) were compared with studies which found VR to be ineffective when compared with 'traditional' treatment methods. To allow for a clear comparison, studies which were equally effective using 'traditional' methods were excluded from this analysis.

### Risk of bias

The two reviewers (MD and NL) independently assessed the risk of bias using the Quality Assessment Tool for Quantitative Studies [19]. This tool has been specially developed for public health research and assesses six components of bias and quality; these include selection bias, study design, confounders, blinding, data collection methods, withdrawals and dropouts. The inter-rater reliability between the authors, using Cohen's Kappa was high (80% agreement, p<.001). Any disagreements between the two reviewers were resolved through discussion or by consulting a third reviewer (JM). The results of the quality analysis were further tabulated to identify any types of bias common to the included studies.

## RESULTS

The study selection process and a summary of the included studies will be presented first, followed by a general overview of the quality of the included studies. Next the main results will be presented, according to the two review objectives; 1) how CBT has been used in VR to treat mental health disorders and 2) which are the treatment characteristics that are linked to effective and ineffective trials.

### Selection and inclusion of studies

**Figure 1:**
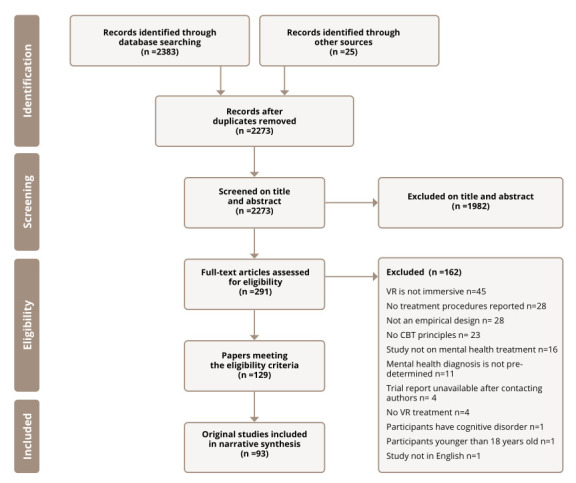
Figure 1. PRISMA flow diagram of the study selection process

Once duplicates were removed, the search generated 2273 references, of which 129 papers met the review inclusion criteria. The 129 papers reported on 93 separate studies; 36 papers reported follow-up data or secondary data analysis of the original 93 included studies. The 36 papers were combined with their original studies and analysed together. The most common reason for exclusion was the use of non-immersive technology (e.g., studies using computer screens). See Figure 1 for the PRISMA flow diagram.

### Characteristics of included studies

Anxiety-related disorders were the most frequently studied group (n=80), followed by eating disorders (n=6), psychosis (n=3), substance disorder (n=3) and finally, one study relating to depression. The majority of the studies were randomized control trials (n=48), followed by cohort studies (n=27), non-randomized clinical trials (n=8) and other designs (n=9). The average sample size across the studies was 41 (range 4 -162), (M=40.7, SD=35.9, n=93).

**Table 1:** Table 1. Breakdown of the quality assessment

Quality assessment	Selection bias rating	Study design rating	Confounding variable rating	Blinding rating	Data collection rating	Withdrawal rating
Strong	[20]	[21]	[22]	[8]	[23]	[24]
Moderate	[25]	[26]	[5]	[16]	[18]	[18]
Weak	[27]	[1]	[13]	[28]	[29]	[30]
Mode	Weak	Strong	Strong	Weak	Strong	Strong

### Quality assessment and risk of bias in included studies

The quality of the studies in this review was found to be predominately weak (see Appendix В for individual study quality assessment). A table was formulated to explore why studies were often of poor quality (Table 1).

The poor quality of the studies can be attributed to selection bias. Most studies either did not report where they recruited their patients from, or they recruited volunteers through advertising. This may have resulted in lower than anticipated drop-out rates, as volunteers might have been more willing to participate. Furthermore, although logistically difficult, most studies did not blind the patients or the assessors to the treatment intervention. This may have resulted in assessment bias.

A cross-tabulation between the quality of studies and the year of publication showed that the quality of studies has not improved over time.

### How has CBT been used in VR to treat mental health disorders?

To address the primary aim of the review, the common characteristics of treatments will be described. For a summary of the treatment characteristics, please view the second column of Table 2.

VR has generally been used as a component in a more extensive treatment protocol (n=58). On average, patients were offered eight treatment sessions, and six of these sessions involved VR technology. The first and the last sessions were psychoeducational, e.g., identifying symptoms and discussing relapse prevention [31]. Treatment was usually delivered once a week for an average of 78 minutes. The average duration of the VR component in these sessions was 53 minutes.

VR treatment was primarily delivered using an HMD device. In all the studies, patients were treated individually in the virtual environment. The VR treatment was generally delivered by therapists (n=38), although only nine studies provided details on the clinical training of the therapist, which included graduate and postgraduate therapists.

The majority of the studies did not report the location of the therapy, however, where the location was explicitly stated, treatments were generally administered in a therapists office/clinic. A typical VR treatment session would involve the patient wearing an HMD, connected to a computer, which is controlled by the therapist.

Virtual reality exposure therapy (VRET) was the most frequently delivered CBT treatment (84 of the 93 studies). During VRET, patients are gradually exposed to a virtual environment that provokes anxiety, e.g., a battlefield in the case of patients with post-traumatic stress disorder [32], or exposure to a spider for patients with arachnophobia [31]. The aim is that patients become desensitized to the fear-provoking stimuli with gradual exposure.

VRET was the most commonly used treatment in this review. Across the nine remaining studies, there was some variation in the definitions used to describe the CBT treatments, e.g., VR enhanced CBT, VR cognitive therapy and repeated behavioural experiment tests. These treatments will be discussed together. Similar to VRET, these treatments all used VR to expose patients to specific, anxiety-provoking virtual environments. However, unlike VRET, the aim of exposure was not just to desensitize the patient to a situation, but to trigger certain emotions or behaviours that therapists can subsequently work on with the patient. For instance, in an eating disorder study, patients were exposed to virtual environments that were thought to trigger emotions related to weight, e.g., restaurants, clothes shopping and a swimming pool. In these environments, patients performed virtual tasks such as weighing themselves and trying on clothes, whilst the therapist discussed feelings and beliefs [33]. Similarly, Pot-Kolder et al. (2018) [30] used VR to expose patients with persecutory delusions and paranoid ideation to stressful social environments, that could trigger fear and paranoid thoughts, e.g., being on the underground or in a cafe. In these virtual environments, they explored and challenged the patient's suspicious thoughts and safety behaviours, and tested harm expectancies.

One study [34] used automated, repeated behavioural experiments for the treatment of a fear of heights. In the virtual environment, patients were guided by a virtual coach to explore and perform height-related tasks (e.g., saving a cat from a high level). In doing this, patients explored how safe they felt at certain virtual heights and often found that they felt safer than they expected.

### What are the treatment characteristics that are linked to effective and ineffective trials?

Studies included in this review varied as to their primary aim; not all the studies investigated or reported treatment effectiveness. Therefore, this section will first provide an overview of all the aims (n=93) then it will specifically focus on the subgroup of studies that aimed to investigate treatment effectiveness (n=62).

**Table 2:**
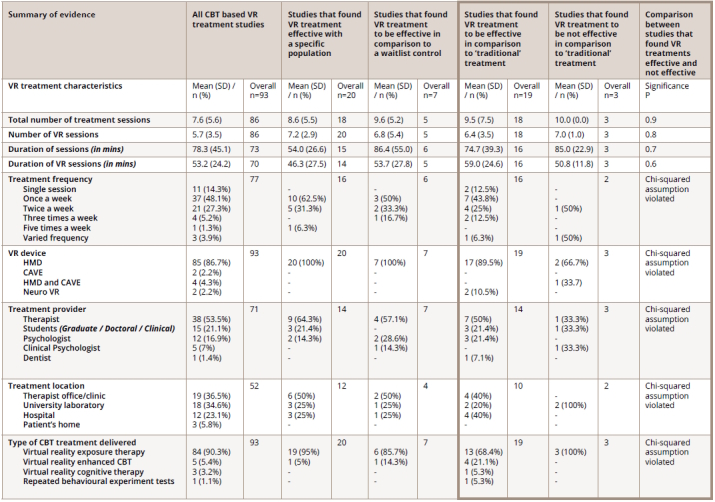
Table 2. Summary of evidence, VR treatment characteristics

**Table 3:** Table 3. Summary of the study aims (Key* studies included in the analysis of the second aim)

Aim (n=93)	Studies that have found VR treatments to be effective (n=46)	Studies that have found VR treatments not effective in comparison to control groups (n=3)	Studies that have found no significant difference between VR treatments and control groups (n=13)	Studies that have not focused on treatment effectiveness (n=31)
*Effectiveness of VR treatment with a specific patient population (n=20)	[32] [25-27,42-57]	No studies	No studies	Not applicable
*Effectiveness of VR treatment in comparison to waiting list (n=8)	[23,24,41,58-61]	No studies	[62]	Not applicable
Importance of presence in VR treatment (n=3)	Not applicable	Not applicable	Not applicable	[21,63,64]
Cost-effectiveness of VR treatment (n=1)	Not applicable	Not applicable	Not applicable	[6]
*Effectiveness of VR treatment in comparison to ‘traditional’ treatment methods (n=34)	[22,28,36-38,40,65-78]	[79-81]	[31] [35,82-91]	Not applicable
Whether VR treatment is enhanced with additional variables (n=21)	Not applicable	Not applicable	Not applicable	[39,92-111]
Patient preference and acceptability (n=2)	Not applicable	Not applicable	Not applicable	[112,113]
Feasibility of VR treatment with cheap consumer hardware (n=1)	Not applicable	Not applicable	Not applicable	[114]
The ability to conduct remote therapy using VR technology (n=3)	Not applicable	Not applicable	Not applicable	[115-117]

The aim of the papers correlated with the year of publication, demonstrating that earlier studies tended to focus on assessing the efficacy of VR treatments, whereas later studies aimed to assess the use of cheaper technology and remote treatment delivery. Fora summary of the study aims, please see Table 3.

### Efficacy of VR treatment within a specific patient population

Of the 20 studies that used a repeated measures design to investigate the efficacy of VR treatment with a specific patient population, all considered VR to be an effective treatment for anxiety-related disorders [119 – 121], substance disorders [45] and eating disorders [118]. For instance, a cohort study comprising 20 combat-related PTSD patients reported post-intervention, that following VRET, 80% of the patients no longer met the criteria for PTSD [32]. Another cohort study with 48 nicotine-dependent adults reported that VR cue exposure treatment reduced the patients’ cigarette cravings [46]. Riva et al. (2002) [118] also used a cohort design with 57 obese and binge eating disorder patients and reported that VR-enhanced CBT, improved patients' body satisfaction.

A breakdown of the treatment characteristics in studies that found VR treatment effective within a specific population, can be found in Table 2. These studies generally consisted of a small sample size (M=25.4, SD=26.8, n=20). The treatments involved a mean of nine sessions, and VR was used in seven of these sessions. The treatment was delivered once a week for a mean duration of 54 minutes.

### Efficacy of VR treatment by comparison with waiting list

Similar to studies that investigated the effectiveness of VR treatment within a specific patient population, the majority of the studies reported VR treatments to be relatively more effective than waiting list controls (n=7). A controlled clinical trial with 23 arachnophobia patients, reported that VRET was effective in treating this phobia. Eighty-three per cent of the patients in the VRET group showed a significant clinical improvement by comparison with no improvement in the waiting list group [58]. An RCT, with 116 psychotic disorder patients, found that VR-CBT did not increase the length of time patients spent with other people, however, it did significantly improve patients' momentary paranoid ideation and anxiety. These improvements were maintained six months after completion of follow-up treatments [30].

Only one RCT that had 32 general anxiety disorder patients, reported that a single session of VRET was not significantly effective by comparison with the waiting list group [62].

The fourth column of Table 2 presents a breakdown of treatment characteristics in the studies that found VR treatment to be more effective than a waiting list control group. The treatment characteristics were similar to those studies that were investigating the effectiveness of VR within a specific population. For instance, studies were delivered across a mean of 10 sessions, and seven of these involved VR.

**Table 4:** Table 4. Summary VR treatment effectiveness in comparison to other 'traditional' treatments

Effectiveness of VR treatment in comparison to ‘traditional’ treatment methods (n=34)	VR treatment less efficacious (n=3)	VR treatment equally efficacious (n=12)	VR treatment more efficacious (n=19)
In-vivo exposure (n=11)	[79,80]	[17,31,35,82,85,90]	[40,75,119]
CBT (n=7)	—	[86,120]	[28,72,74,76,121]
Imaginal exposure (n=3)	—	[122,123]	[77]
Psychoeducation (n=2)	—	—	[124,125]
Treatment as usual (n=2)	—	—	[34,70]
Bibliotherapy (n=1)	—	—	[109]
Prolonged exposure (n=1)	[81]	—	—
Integrated psychological therapy (n=1)	—	—	[38]
Nicotine replacement (n=1)	—	—	[66]
Information pamphlet (n=1)	—	—	[22]
Control exposure (n=1)	—	[88]	—
Attention placebo (n=1)	—	—	[126]
Computer-aided exposure (n=1)	—	[127]	—
Relaxation group (n=1)	—	—	[71]

### Effectiveness of VR treatment by comparison with 'traditional' treatment methods

The majority of the studies in this review aimed to identify the effectiveness of VR treatments by comparison with 'traditional' treatment methods. Thirty-one out of the 34 studies (91.2%) considered VR treatments to be equally or more efficacious than traditional treatment methods. See Table 4 for a summary of VR treatment effectiveness by comparison with other 'traditional' treatments.

Three RCTs considered VR treatments to be less efficacious than 'traditional' treatment methods. Two compared the effectiveness of VRET with in vivo exposure treatment, where patients are physically exposed to the feared stimuli. Meyerbroeker et al. (2013) [80] randomized 55 agoraphobia patients and found that in-vivo exposure decreased patients' panic severity more than VRET. Similarly, Kampmann et al. (2016) [79] randomized 60 patients with a social anxiety disorder and noted that in-vivo exposure decreased patients' social anxiety symptoms. Another RCT compared the effectiveness of VRET with prolonged exposure in 162 combat-related PTSD patients. Follow-ups at three and six months reported that prolonged exposure had significantly reduced more PTSD symptoms than VRET [81].

The number of studies that reported negative results is minimal (n=3). Despite the small number of negative results, studies between effective and ineffective VR treatments were compared using a t-test. Studies which found VR treatment to be inferior to traditional methods had a larger sample size (M=92.3) than those which considered VR treatments to be superior (M=48.8). However, this difference was not significant (T=-1.8, DF=20, P=0.09).

Data were also collected in relation to participant drop-out rates. The patients' reasons for dropping out of VR treatments included VR exposure not arousing the anxiety that is necessary for desensitization [78], VR causing motion sickness and conflicts with patients' diaries [126]. The patients' reasons for dropping out of 'traditional' treatments included not wanting in-vivo exposure [40], not being satisfied with the treatment allocation and wanting to pay for VR therapy [75]. Studies which found VR treatments more effective than 'traditional' treatments reported significantly lower VR drop-out rates (M=15.1%) than treatments which regarded 'traditional' treatments as superior to VR treatments (M=39%) (T=-2.4, DF=13, P=0.04).

Therefore, patient drop-out rates were a variable in the success of VR treatment. The other treatment variables, such as the number and duration of the sessions, were very similar across the two outcomes. Please see Table 2, Column 6 for comparisons between the variables.

## DISCUSSION

### Main findings

VR has mainly been used in the treatment of anxiety-related disorders, and treatment has usually taken the form of exposure therapies. VR has generally been used as a component in a more extensive treatment protocol. On average, patients were offered eight sessions of therapy, and six of these sessions involved VR technology. The sessions were usually delivered once a week for an average of 53 minutes.

Even though the overall quality of the evidence is weak, VR treatments seemed to perform comparably in terms of efficacy with 'traditional' face-to-face treatments. Treatment characteristics, such as the number and duration of sessions, were very similar between studies that regarded VR treatment as effective and those that found it not to be effective. However, patient drop-out rates were significantly lower in studies that considered VR treatment to be effective by comparison with those that found it ineffective.

### Comparison with literature

This review is the first to investigate how VR has been used in CBT (a psychotherapeutic approach) to treat a variety of mental health disorders. Previous VR reviews have focused on providing a general overview of the field [8] or reported treatment outcomes for specific diagnoses [2].

Results from this review support the findings from previous reviews, that VR is an acceptable and promising therapeutic tool for mental health treatment [4]. It can be used to deliver cognitive rehabilitation, social skills training interventions and VR-assisted therapies for psychosis [5]. VRET is equally effective as in-vivo exposure for the treatment of anxiety-related disorders [3].

However, regardless of the wide variety of CBT treatment techniques and applications, research into VR treatments still focuses primarily on treating anxiety-related disorders with exposure-based therapies [8,128-132]. There is still limited research into different types of CBT therapies, e.g., group therapies, and limited research into applications for different diagnostic groups, e.g., patients with mood disorders [8].

In a recent review, Freeman et al. (2017) [8] highlighted evidence that drop-out rates may be lower with VR treatments. This review supports this finding; overall, fewer patients dropped out of VR treatments than 'traditional' treatments. However, similar to Freeman's review, as differences in drop-outs may have been due to the quality of face-to-face treatments, this review is also unable to make any firm conclusions regarding these differences, but it does highlight the importance of offering high-quality treatments in research studies.

### Strengths and Limitations

This review is the first to collate data as to how CBT has been used in VR to treat mental health disorders. The shared treatment characteristics (e.g., eight sessions, once a week for approximately one hour) identified in this review, could potentially prevent researchers from wasting resources developing one-off interventions. Building on the shared treatment characteristics identified in this review, may potentially enable researchers to explore new VR treatment methods or explore VR treatments for under-researched diagnoses.

The treatment framework developed from this review (e.g., eight sessions, once a week for approximately one hour using an HMD) may have potential clinical implications. The lack of VR treatment guidelines could potentially have been a barrier to VR treatments entering mainstream clinical practice. Building on the treatment framework developed from this review, therapists or clinics may feel more confident to offer their patients CBT-based VR treatments.

The results from this review need to be understood within the context of its limitations. This review consisted of a high volume of papers, produced from original studies. Many authors used data across different studies, and some authors avoided referencing their data source. This has made the separation of the original studies from secondary analysis papers difficult. A significant amount of time was spent matching the papers, and studies were compared for similarities and differences. Therefore, although the potential risk of over representation of some studies is minimal, this cannot be ruled out completely. However, as the results were mainly synthesized narratively, according to treatment characteristics and methods, this would have had a limited impact on the findings.

The second aim of this review was to report the shared characteristics of effective and ineffective CBT methods. Even though the review search criteria and strategy were extensive, this review may have been affected by publication bias. Negative results are less likely to have been published. This review only identified three recent studies that reported the inferiority of VR treatment. This may be an indicator of time-lag bias, where positive findings are published first and negative findings later. Therefore, the results section of this review, comparing the shared treatment characteristics of effective and ineffective CBT methods, should be interpreted with caution.

Furthermore, this review has only conducted causal associations but has not tested these associations in a formal manner, e.g., this review cannot conclude that reducing the number of sessions from eight to five will reduce treatment effectiveness. However, the analysis conducted in this review and the framework created, is based on the best available evidence, although future studies would be required to test the framework generated.

## CONCLUSIONS

This review is the first to synthesize CBT treatment characteristics and methods used in VR to treat mental health disorders. The shared treatment characteristics of a total of eight treatment sessions, once a week for approximately an hour, could be used as a treatment template by future researchers. This could potentially prevent researchers from spending time and money developing their own one-off interventions. Furthermore, it may possibly enable researchers to explore new VR treatment methods or explore VR treatments for under-researched diagnoses.

## Acknowledgements

The authors wish to acknowledge the Faculty Liaison Librarian, Paula Funnell for assisting with the search strategy of this study. The East London Foundation Trust whose funding supported this research and the researchers at the Unit for Social and Community Psychiatry, who provided feedback on an early draft of this paper.

## Author Contributions

Merve Dilgul: conceptualization, methodology, formal analysis, writing-review and editing; Jasmine J. Martinez: formal analysis; Neelan Laxhman: formal analysis; Stefan Priebe: conceptualization, methodology, supervision; Victoria Bird: conceptualization, methodology, writing-review and editing, supervision.

## Funding

The East London NHS Foundation Trust has funded this study as part of a PhD Project. The funder of the study had no role in study design, data collection, data analysis, data interpretation or writing of the report. The corresponding author had full access to all the data in the study and took the final decision to submit for publication.

## Conflict of Interest

The authors report no conflict of interest.
